# Construction and validation of content of one instrument to assess falls in the elderly

**DOI:** 10.1590/S1679-45082018AO4154

**Published:** 2018-06-05

**Authors:** Michele Bittencourt Silveira, Ricardo Pedrozo Saldanha, José Carlos de Carvalho Leite, Thamyres Oliveira Ferreira da Silva, Thiago Silva, Lidiane Isabel Filippin

**Affiliations:** 1 Universidade La Salle, Canoas, RS, Brazil.

**Keywords:** Validation studies, Surveys and questionnaires, Aging, Accidental falls, Public health, Estudos de validação, Inquéritos e questionários, Envelhecimento, Acidentes por quedas, Saúde pública

## Abstract

**Objective:**

To develop and validate the content of the online Questionnaire for Fall Risk Assessment in the Elderly.

**Methods:**

The instrument was developed based on the International Classification of Functioning, Disability and Health (ICF) of the World Health Organization. Initially, the set of items was submitted to evaluation of judges (healthcare professionals with experience in elderly health), who could suggest inclusion or exclusion of questions from the instrument; they were also asked to rate each question according to the expected scope. At this stage, clarity and relevance levels for each item were evaluated, generating a total of Content Validity Coefficient (C_t_VC).

**Results:**

Content Validity Coefficient values were satisfactory for both clarity (C_t_VC=0.76) and relevance (C_t_VC=0.82) of the questions. Next, a group of elderly volunteers participating in a socializing group evaluated the questionnaire for comprehension. The level of comprehension for each item was identified on a Likert scale, ranging from 0 to 5. The questionnaire was considered easy to understand by most participants (95%), with a mean of 4.75 (±0.11) points for each item.

**Conclusion:**

The instrument showed acceptable psychometric qualities for screening fall risk among the elderly population. Future studies shall investigate different validation aspects of construct for this measure.

## RESUMO

**Objetivo:**

Desenvolver e validar o conteúdo do Questionário *on-line* para Avaliação do Risco de Quedas em Idosos.

**Métodos:**

A construção do instrumento foi baseada na Classificação Internacional de Funcionalidade, Incapacidade e Saúde (CIF) da Organização Mundial da Saúde. Inicialmente, o conjunto de itens foi submetido à avaliação de juízes (profissionais da saúde com experiência em saúde do idoso), que puderam sugerir questões a serem incluídas/excluídas do instrumento, além de verificarem cada questão do questionário, observando se as dimensões a serem avaliadas tinham sido abrangidas. Nesta etapa, os níveis de clareza e pertinência do conteúdo foram avaliados, gerando um total de Coeficiente de Validade de Conteúdo (CVC_t_).

**Resultados:**

Os valores do Coeficiente de Validade de Conteúdo foram satisfatórios, tanto para a clareza (CVC_t_=0,76) quanto para a pertinência (CVC_t_=0,82) das questões. A seguir, a compreensão do questionário foi avaliada por idosos voluntários participantes de um grupo de convivência. O grau de compreensão para cada questão foi identificado em uma escala do tipo Likert, variando de 0 a 5. O questionário foi considerado de fácil compreensão pela maioria dos idosos (95%), com média de 4,75 (±0,11) pontos para cada questão.

**Conclusão:**

O instrumento apresentou qualidades psicométricas aceitáveis para ser utilizado como ferramenta para rastrear o risco de quedas entre a população de idosos. Futuros estudos devem investigar diferentes aspectos da validade de construto desta medida.

## INTRODUCTION

There has been an exponential increase in the number of elderly individuals in the last decades, both in developed and developing countries. In Brazil, the number of elderly people is expected to triple by 2050.^(^ ^1^ ^)^ In this context, geriatric healthcare is becoming more and more prominent, with an increasing number of scientific research in the field of Gerontology. Chronic diseases and their multidimensional impact on health and quality of life of elderly patients are a major concerns in this age group. Falls are very prevalent in this population and affect their functional capacity, autonomy, social life, and healthcare needs; therefore, falls deserve attention of healthcare professionals who assist the elderly.^(^ ^2^ ^,^ ^3^ ^)^

Falls are considered a health indicator and a public healthcare issue among the elderly, especially older individuals.^(^ ^4^ ^)^ According to the World Health Organization (WHO), the annual global fall rate ranges from 28 to 35% among individuals aged over 65 years, and this prevalence increases to 32 to 42% among those aged over 70 years.^(^ ^1^ ^)^

According to the Brazilian Ministry of Health, the Unified Health System (SUS - *Sistema Único de Saúde*) spends over R$51 million in treatments related to falls, per year.^(^ ^5^ ^)^ Falls have a multifactorial etiology, and are often associated to a decreased functional capacity, unstable gait and loss of balance resulting from sensorineural loss and osteoarticular deficits in the elderly. The main consequences of falls in the elderly include fractures; increased risk of death; fear of falling again, which leads to social isolation and restriction of Activities of Daily Life; general health decline; and increased institutionalization.^(^ ^6^ ^,^ ^7^ ^)^

Considering the impact falls have in elderly health, it is important to identify risk factors and use validated instruments to assess fall risk in this population, especially when it comes to prevention. There are currently some instruments for fall risk assessment, but few have been prepared and validated in a way that could be self-applicable to the aging population in Brazil.^(^ ^8^ ^,^ ^9^ ^)^ Questionnaires are important tools to obtain information, especially in population studies, and they must present psychometric qualities (validity and reproducibility) to ensure reliability of the indicators assessed.^(^ ^3^ ^)^

To that end, instrument validity (including content, criteria and construction) is extremely relevant because it determines if the instrument is in fact able to evaluate the measures for which it was designed.^(^ ^10^ ^)^ Validity of a questionnaire content - one type of validation used in this study, is subjectively judged by specialists in the field to determine if the questionnaire can represent the behavior of the sample being evaluated. This stage checks if the item representativeness adequately expresses the content to be evaluated.^(^ ^11^ ^)^ It is an important stage in the development of a new instrument.

That means this type of evaluation determines if the instrument’s contents effectively explore (with clear language and theoretical relevance) the items to measure a certain phenomenon to be investigated.

Considering the exponential growth of the elderly population, health problems caused by falls, and the lack of self-applicable questionnaires for fall risk assessment among the elderly, it is important to build instruments for this population that yield a more efficient and generalized understanding of older individuals. The objective of this tool is to improve safety and quality of health of elderly individuals, and to aid healthcare professionals in the evaluation and guidance in fall risk management.

## OBJECTIVE

To establish and test the metric principles of content validity of an instrument to assess falls in the aged.

## METHODS

This is a methodological study developed in three stages: instrument construction, Content Validity Coefficient (CVC), and verbal comprehension.

### Stage I – instrument construction

The first stage - construction of the online Assessment Instrument for Elderly Falls (IAQI - *Instrumento de avaliação de quedas para idosos*) – was based on the WHO International Classification of Functioning, Disability and Health (ICF).^(^ ^12^ ^)^ The ICF is a useful tool to describe and understand an individual’s health status and to identify contextual factors (environmental and personal) that favor their functionality. The ICF is widely used, including for population surveys on health and disabilities -it proposes a conceptual functionality and disability model, theoretically divided into two factors: (a) functionality and disability; and (b) contextual factors. Thus, in this study, each group of questions was divided into two components, in addition to domains, constructs, and positive and negative aspects proposed by the ICF (Appendix 1).

The IAQI was constructed in two blocks. The first focused only on identification and sociodemographic data, and the second block had eight questions – questions 5, 7, and 8 had unfolding questions for positive answers, bringing us to a total of 14 questions about the outcome (fall risk).

Nine questions were related to functionality and disability factors and five were about contextual factors. For the former, we approached the domains and constructs that make up the components of body functions and structures, and activity and participation. For contextual factors, we addressed only domains and constructs inside the environmental factor component.

After preparing the first version, the instrument was submitted to content evaluation (clarity and relevance of questions), and the processes are described below:

### Stage II – content validity

For this stage, we used the CVC procedure described by Hernandez-Nieto, in 2002.^(^ ^13^ ^)^ The CVC was created to more adequately meet the needs of this kind of validity. It is recommended that item/question evaluators be three or five experts, with recognized theoretical and practical knowledge in the specific area. Content validity is calculated through the CVC because it is an ordinal scale.

Three evaluating judges participated in this stage. Inclusion criteria of the judges were: subject expertise and at least 10-year professional experience; Masters or Doctorate degree in the field of health; teaching or delivering care in public health. Two evaluators were lecturers and had worked in Public Health for 9 years; the third evaluator had a major in physical education and a Master’s Degree in Human Aging (concluded four years before).

The material was sent electronically to the judges, with a request that it be returned within 30 days. The criteria used by the judges to evaluate the questions were clarity and relevance, presented on a Likert scale (graded from 1 to 5). The judges were asked the following questions:^(^ ^14^ ^,^ ^15^ ^)^ regarding clarity (CVC) – “Do you believe this item/question is clear enough to be understood by the target population?”; and, regarding relevance (CVC) – “ Do you believe this item is relevant to the study and the target population?” Regarding clarity, the questions could be scored from very unclear (1) to very clear (5). In the relevance scale, the questions could be scored from very irrelevant (1) to very relevant (5). In addition to the two scales, the judges could add a qualitative evaluation next to each question for further improvement.^(^ ^15^ ^)^

Based on these answers, we were able to calculate the instrument CVC. This calculation is done in five stages. The first, based on the judge grades (1 to 5), is the calculation of the mean grade given to each item (M_x_). The second stage, with base on the mean grade (M_x_) is the calculation of the initial CVC for each item (CVC_i_) divided by the maximum relevance or clarity grade (5). The third stage is the calculation of error (Pe_i_) to discount possible biases of the judges for each question. In this case, one (1) is divided by the number of evaluating judges, to the power of the same number of evaluators. With that, the final CVC (fourth stage) of each item/question (CVC_c_) can be calculated by subtracting; *i.e*, Pe_i_ – CVC_i_ = CVC_c_. The final stage is the calculation of the total CVC of the questionnaire (CVC_t_) for each characteristic (language clarity, practical and theoretical relevance) by subtracting the mean Pe_i_ (MPe_i_) from the mean CVC_i_ (MCVC_i_). After calculations, the literature^(^ ^16^ ^-^ ^18^ ^)^ considers acceptable questions with a CVC_t_ between 0.7 and 0.8).^(^ ^19^ ^)^

### Stage III – verbal comprehension

The final version of IAQI was used to test the clarity of questions and facility to answer in a group of elderly individuals, aiming to identify possible comprehension issues.

For this stage, we interviewed 24 elderly individuals (≥60 years), participants of a social group in the city of Porto Alegre (RS), Brazil. They were invited and agreed to participate in this stage of the study after its method and objective were clarified. While applying the questionnaire to the participants, the interviewer was in charge of observing and recording any comprehension difficulties for each question and the total interview time.

Each question received an answer using the Likert scale (0 to 5) to evaluate the level of comprehension. It was established that scores 0, 1, 2, and 3 would indicate insufficient comprehension.^(^ ^20^ ^)^ To analyse IAQI level of comprehension by the elderly, we calculated mean values, with their respective standard deviations (SD), of answers from the Likert scale using the software Statistical Package of the Social Sciences (SPSS), version 21.0.

This study was analyzed and approved by the Research Ethics Committee of the *Universidade La Salle*, in Canoas (RS), Brazil, under protocol no. 642.310, CAAE: 30236314.0.0000.5307.

## RESULTS

Instrument to assess falls in the elderly was analysed based on two criteria: language clarity and relevance. Table 1 presents the questions referent to the second block (fall risk established based on the ICF) that is part of the initial version of the questionnaire.

**Table 1:** Initial version of the online Assessment Instrument for Elderly Falls

Question	Description of items/questions
1	Do you feel:
	a) Dizziness or loss of balance when standing or walking
	b) Muscle weakness (in your whole body)
	c) Muscle weakness (only in your arms)
	d) Muscle weakness (only in your legs)
	e) Difficulty to walk
	f) Tiredness or exhaustion
2	In the last 12 months, do you feel your strength has decreased?
3	Do you feel your gait has become slower in the last year, in comparison to the year before that?
4	Do you use any device to move/walk?
5	Do you do any physical activity? (regularly – twice a week, for 30 minutes)
	If the answer for question 5 is “yes”, the interviewee is asked how long he/she has been doing physical activities.
5.1	How long have you been doing physical activities?
	a) Less than one year
	b) 2 to 3 years
	c) 4 to 5 years
	d) More than 6 years
6	Do you think your physical activity has decreased in comparison to last year?
7	Have you lost weight in the last year without having gone on a diet?
	If the answer to question 7 is “yes”, the interviewee is asked how many kilos he/she has lost.
7.1	a) Lost 1kg to 3kg
	b) More than 3kg
	c) Has not lost weight
8	Have you fallen in the last 12 months?
	If the answer to question 8 is “yes”, the interviewee is asked four more questions about the fall. If the answer to question 8 is “no”, the questionnaire is over.
8.1	How many times?
	a) once
	b) twice
	c) 3 times
	d) More than 3 times
8.2	Place of the fall:
	a) At home (bedroom, bathroom, kitchen, living room)
	b) On the street (sidewalk, steps, work)
	c) In the backyard (yard, garden)
8.3	Type of fall:
	a) From standing (was standing and fell)
	b) Down a staircase
	c) From sitting (was sitting down or in the act of sitting and fell)
	d) Other
8.4	Due to fall, you:
	a) Had to be admitted to the hospital
	b) Suffered a fracture (broken bone)
	c) Suffered a sprain or dislocation (moved out of the normal position)
	d) Had difficulty walking after the fall
	e) Became dependent on someone else to perform daily activities
	f) Are afraid to fall again

In this version of the instrument, 28.5% of questions presented CVC_c_ scores below 0.70 regarding clarity. Approximately 57.1% presented results between 0.70 and 0.80, and only 14% of questions had CVC_c_ scores above 0.80. The items/questions with the lowest scores in the clarity evaluation were CVC_c_ = q1=0.56; q5=0.63; q5.1=0.36; q8.4=0.69 (Table 2).

**Table 2:** Content Validity Coefficient, first assessment

CVC – Clarity					CVC – Relevance				
	
Item	Mean	CVC_i_	Pe_i_	CVC_c_	Item	Mean	CVC_i_	Pe_i_	CVC_c_
1	3	0.6	0.04	0.56	1	4	0.8	0.04	0.76
2	3.66	0.73	0.04	0.70	2	4	0.8	0.04	0.76
3	4	0.8	0.04	0.76	3	4.33	0.86	0.04	0.83
4	3.66	0.73	0.04	0.70	4	4	0.8	0.04	0.76
5	3.33	0.66	0.04	0.63	5	4.33	0.86	0.04	0.83
5.1	2	0.4	0.04	0.36	5.1.	4.66	0.93	0.04	0.90
6	3.66	0.73	0.04	0.70	6	4	0.8	0.04	0.76
7	4	0.8	0.04	0.76	7	4	0.8	0.04	0.76
7.1	4.66	0.93	0.04	0.90	7.1.	4.66	0.93	0.04	0.90
8	4	0.8	0.04	0.76	8	5	1	0.04	0.96
8.1	4.66	0.93	0.04	0.90	8.1.	5	1	0.04	0.96
8.2	3.66	0.73	0.04	0.70	8.2.	4	0.8	0.04	0.76
8.3	3.66	0.73	0.04	0.70	8.3.	3.66	0.73	0.04	0.70
8.4	3	0.6	0.04	0.56	8.4.	4.33	0.86	0.04	0.83
					
	Total (CVC_t Clarity_)			0.69		Total (CVC_t Relevance_)			0.82

CVC_i_: content validity coefficient for each item; Pe_i_: error; CVC_c_: final content validity coefficient; CVC_t_: total content validity coefficient.

Regarding relevance, the result were distributed as follows: 50% <0.70; 0% between 0.70 and 0.80; and 50% >0.80. In comparison to clarity, relevance had better results in the same questions: q1=0.76; q5=0.83; q5.1=0.90; q8.4=0.83 (Table 2).

Considering CVC_c_ values and observations made, a second round of evaluations was sent to the judges. In this case, a new form was sent containing only the criterion of clarity, because of its previous result of CVC_t_ under 0.70. Table 3 presents the CVC values in the second evaluation after readjustment of the questionnaire. After the second evaluation of clarity, 21.4% of questions had CVC_c_ scores under 0.70; about 28.1% had scores between 0.70 and 0.80; and 50% had CVC_c_ results above 0.80.

**Table 3:** Calculation of the Content Validity Coefficient, as per clarity and relevance of questions after final evaluation of the judges

	CVC - Clarity _(version 2)_			
	
Item	Mean	CVC_i_	Pe_i_	CVC_c_
1	4.33	0.866	0.04	0.83
2	4.33	0.866	0.04	0.83
3	4	0.8	0.04	0.76
4	4.66	0.93	0.04	0.90
5	3.66	0.73	0.04	0.70
5.1	4.33	0.86	0.04	0.83
6	4.33	0.86	0.04	0.83
7	4	0.8	0.04	0.76
7.1	4.33	0.86	0.04	0.83
8	4.33	0.86	0.04	0.83
8.1	4	0.8	0.04	0.76
8.2	3.33	0.66	0.04	0.63
8.3	3	0.6	0.04	0.56
8.4	3.33	0.66	0.04	0.63
		
	Total - CVC_t Clarity (version 2)_			0.76

CVC_i_: content validity coefficient for each item; Pe_i_: error; CVC_c_: final content validity coefficient; CVC_t_: total content validity coefficient.

After all suggestions proposed by the judges, it was possible to calculate the CVC_t_ for clarity, with a result of 0.76, which is considered above the cut-off point suggested in the literature.^(^ ^19^ ^)^ Regarding relevance, results were better – 50% of questions received a CVC evaluation above 0.80. Total content validity coefficient for relevance was 0.82 (Table 3). Based on the CVC calculation (total score - CVC_t_), the final version was elaborated and is presented in table 4.

**Table 4:** Final version of the online Assessment Instrument for Elderly Falls

Question	Description of items/questions
1	In the last month, have you felt (you may mark more than one answer):
	a) Dizziness when standing or walking
	b) Weakness in your whole body
	c) Weakness only in your arms
	d) Weakness only in your legs
	e) Difficulty to walk
	f) Tiredness or exhaustion
2	In the last year, do you feel your strength has decreased when performing simple tasks, *e.g.*, climbing stairs or steps, opening containers, carrying grocery bags, cleaning or tidying the house?
	( ) Yes
	( ) No
3	Do you feel the way you walk has become slower in the last year, in comparison to the year before that?
	( ) Yes
	( ) No
4	When you walk, do you need some form of support or device (for example: cane, crutches, or help of another person)?
	( ) Yes
	( ) No
5	Do you exercise regularly, for example: walking, cycling, going to the gym, doing aerobics, playing soccer or volleyball, at least twice a week for at least 30 minutes each day?
	( ) Yes
	( ) No
	If the answer for question 5 is “yes”, the interviewee is asked how long he/she has been exercising.
5.1	How long have you been exercising regularly (that is, without stopping for any period of time)?
	a) Less than one year
	b) 1 to 2 years
	c) 2 to 3 years
	d) 3 to 5 years
	e) More than 5 years
6	Do you believe your ability to perform daily tasks, such as walking, going uphill, tidying the house, has decreased in comparison to the previous year?
	( ) Yes
	( ) No
7	In the last year, have you lost weight or have your clothes become loose, without you having changed the amount of food you eat?
	( ) Yes
	( ) No
	If the answer to question 7 is “yes”, the interviewee is asked how many kilos he/she has lost.
7.1	a) Lost between 1kg and 3kg
	b) More than 3kg
8	Did you fall in the last year?
	( ) Yes
	( ) No
	If the answer to question 8 is “yes”, the interviewee is asked four more questions about the fall. If the answer to question 8 is “no”, the questionnaire is over.
8.1	How many times did you fall in this period (last year)?
	a) once
	b) twice
	c) three times
	d) more than three times
8.2	Where were you when you fell (you may mark more than one option if you have fallen more than once):
	a) At home (bedroom, bathroom, kitchen, living room)
	b) In the backyard of your home (yard or garden)
	c) On the street (sidewalk, curb, slippery sidewalk, at work, sports club, at the gym)
8.3	How did you fall (you may mark more than one option if you have fallen more than once)? You:
	a) Were standing and fell
	b) Were walking and fell
	c) Were walking, tripped and fell
	d) Fell down a staircase
	e) Fell from a chair (you were sitting and fell or were in the act of sitting and fell)
	f) Other
8.4	Because of the fall, you (you may mark more than one option):
	a) Had no consequences
	b) Had to be admitted to the hospital
	c) Suffered a fracture (broken bone)
	d) Had difficulty walking after the fall
	e) Have permanent (forever) difficulty to perform daily activities, such as brushing your hair, getting dressed, taking a shower, or eating on your own
	f) Can no longer perform tasks on your own, such as doing the dishes, tidying the house, grocery shopping, cooking for yourself, answering the door, taking a bus or riding in a car)
	g) Are afraid to fall again
	h) Are apprehensive about performing daily activities for fear of falling again

After evaluation by the experts, IAQI verbal comprehension was evaluated by a group of volunteer elderly individuals. Instrument to assess falls in the elderly obtained a mean level of comprehension of 95% (mean±SD = 4.75±0.11). All 24 elderly individuals who participated voluntarily in the instrument pre-test, said the instrument was easy to understand. Only three participants gave four questions a score under 2 (“not very clear”), and the reasons they gave were they did not understand question 7.1 was related to the answer given to question 7, and they also did not understand some questions could have more than one option as an answer.

## DISCUSSION

Healthcare services are facing a challenge to assist the elderly population, which is growing considerably and presents a profile marked by chronic diseases and morbidity. This study proposes the use of a self-applicable, low-cost, quick-to-use instrument that is catered to the elderly population. It also offers educational guidelines to minimize public health issues, which generate several limitations and negatively impact the individuals’ quality of life.

After evaluations done by experts, we were able to improve the questionnaire, and some questions were rewritten and/or sub-items were added to further adjust the tool to its target audience. These adjustments were made mainly to questions that were given low CVC_c_ scores regarding clarity. For example, questions 5 and 5.1 about the regular practice of physical activities and how long the individual has been practicing them. This question was rewritten to distinguish between physical exercise and physical activity. Questions 1 and 8.4 were also adjusted. After the judges evaluated question 1, the answers included loss of balance (dizziness), decreased muscle strength (weakness), difficulty walking and fatigue (tiredness and exhaustion) noticed in the last month. All these answers are related to intrinsic factors related to falls in elderly individuals.^(^ ^21^ ^)^ Delbaere et al.,^(^ ^22^ ^)^ established the psychometric properties of the simple test of choice stepping reaction time (CSRT), investigating its validity and reliability to predict falls in the elderly. CSRT accurately reflects the ability to advance quickly and appropriately to avoid obstacles at the end of a path, which is supported by neuropsychological, balance, and sensory systems that are paramount for balance control. The authors concluded that, for each increased SD in the test, elderly individuals presented 74% more chances of suffering multiple falls, which proved the study usefulness in the evaluation of individuals at risk of falling. However, clinical applicability depends on the training received by the professional who will apply the test. Moreover, elderly patients need to be evaluated by a healthcare professional to better predict the fall risk.

With the respective proposed alterations, question 1 got a better CVC_c_ score for clarity (CVC_1st version_=0.56; CVC_2nd version_=0.83). After readjustment, question 8.4 received a score below the cut-off point suggested by the literature for clarity (CVC – 0.63). Nevertheless, the question was included due to its relevance to the theme and adequate score for relevance (CVC=0.83).

In total, the instrument was finalized with 14 questions, six of which are multiple-choice, and the other eight have simple dichotomous responses (“yes” or “no”).

Chang et al.,^(^ ^23^ ^)^ conducted the psychometric assessment of the psychological and social well-being indicators for elderly residents of Chicago, and observed that the use of short instruments with objective answers in population interviews is more adequate to detect health problems in elderly individuals, especially when the study population presents a low level of education. Since the IAQI, proposed in this study, is self-applicable, the questionnaire was carefully prepared with simple language that could be understood even by those with intellectual challenges. Moreover, the instrument’s layout is catered to the elderly population. Pedreira et al.,^(^ ^3^ ^)^ evaluated the content validity of the Elderly Health Assessment Tool in a low schooling level population in the Southwest region of the Brazilian State of Bahia. The multidimensional questionnaire was composed of seven blocks (sociodemographic information, housing conditions, life habits, functional capacity, health status, mental health, and quality of life), with a total of 207 questions. After content validation by judges with experience in epidemiology of aging research, 50 questions were removed because the questionnaire was deemed too long. Elderly Health Assessment Tool showed a mean level of agreement score of 86% and a content validity index of 93.47%.

Despite presenting appropriate clarity and relevance scores, some of the IAQI questions were revised and altered based on the judges’ comments. Question 2 (about reduced muscle strength) had satisfactory clarity and relevance scores in the first evaluation but, after the judges’ observations, some examples of daily activities were added to aid the interviewee (*i.e*. climbing stairs, opening containers, carrying grocery bags, etc.).

Finally, after the judges’ evaluation, a group of elderly volunteers evaluated the questionnaire verbal comprehension. These data were similar to the findings by Ulian et al.,^(^ ^24^ ^)^ who evaluated verbal comprehension after transcultural adaptation of the State and Trait Food-Cravings Questionnaire to Portuguese, and found a mean comprehension level of 95.4%.

This study has some limitations. Firstly, the evaluating judges were gerontology professionals from the Brazilian state of Rio Grande do Sul, which brings better knowledge about the local reality of these elderly individuals. Secondly, the study presented adequate clarity and relevance scores in the questions presented to evaluate risk of falls; however, other validations are necessary before it can be used in clinical settings.

For clinical uses, further analyses must be conducted, such as construct validity (confirmatory factorial analysis), precision (alpha, omega), and risk measure for each respondent (Receiver Operating Characteristic – ROC - curve). After these procedures, IAQI must be projected in a responsive platform to be adjusted to any kind of device. The unprecedented character of this instrument is the format in which it is being proposed – it is catered to the elderly population and was prepared to be self-applicable and comprehensible to these individuals. It can inform respondents about risk classification and guide them through measures of fall risk reduction.

## CONCLUSION

The total clarity and relevance Content Validity Coefficient was considered adequate. The items that presented clarity indices below literature recommendations were kept for being considered important for value measurement. The questionnaire for assessing fall risk in the elderly is a valid instrument from a content perspective (clarity and relevance items/questions).

## Appendix 1

**Table:** Questions related to the fall risk outcome, based on the organizational system of the International Classification of Functioning, Disability and Health (ICF)

Question	ICF factor	Component	Domain	Construct	Classified block or category
1	Functionality and disability	Body functions and structures	Body functions	Changes in body functions (physiological)	Global mental functions
2	Functionality and disability	Body functions and structures	Body functions	Changes in body functions (physiological)	Global mental functions
3	Functionality and disability	Activity and participation	Vital areas (tasks)	Task performance in a habitual environment	Walking and moving around (mobility)
4	Functionality and disability	Activity and participation	Vital areas (tasks)	Task performance in a habitual environment	Walking and moving around (dexterity)
5	Contextual factors	Environmental factors	External influences on functionality and disability	Facilitating or limiting impact of characteristics of the physical, social, and attitudinal world	Self-care
6	Functionality and disability	Activity and participation	Vital areas (tasks)	Task performance in a habitual environment	Global mental functions
7	Functionality and disability	Body functions and structures	Body functions	Changes in body functions (physiological)	Functions related to the digestive system
8	Functionality and disability	Body functions and structures	Body functions	Changes in body functions (physiological)	Vision and related functions; vestibular hearing functions; global mental functions
8.1	Functionality and disability	Body functions and structures	Body functions	Changes in body functions (physiological)	
8.2	Contextual factors	Environmental factors	External influences on functionality and disability	Facilitating or limiting impact of characteristics of the physical, social, and attitudinal world	
8.3	Contextual factors	Environmental factors	External influences on functionality and disability	Facilitating or limiting impact of characteristics of the physical, social, and attitudinal world	
8.4	Functionality and disability	Body functions and structures; and activities and participation	Body functions; and vital areas	Changes in body functions and task performance in a habitual environment	Specific mental functions; domestic life: main areas of life

## References

[1] World Health Organization (WHO) (2007). Ageing, and Life Course Unit. WHO global report on falls prevention in older age.

[2] Veras R (2009). Envelhecimento populacional contemporâneo: demandas, desafios e inovações. Rev Saude Publica.

[3] Pedreira RB, Rocha SV, Santos CA, Vasconcelos LR, Reis MC (2016). Validade de conteúdo do Instrumento de Avaliação da Saúde do Idoso. einstein (São Paulo).

[4] Chan BK, Marshall LM, Winters KM, Faulkner KA, Schwartz AV, Orwoll ES (2007). Incident fall risk and physical activity and physical performance among older men the osteoporotic fractures in men study. Am J Epidemiol.

[5] Brasil, Ministério da Saúde (2012). Quedas.

[6] Park SH (2018). Tools for assessing fall risk in the elderly: a systematic review and meta-analysis. Aging Clin Exp Res.

[7] Ferretti F, Lunardi D, Bruschi L (2013). Causas e consequências de quedas de idosos em domicílio. Fisioter Mov.

[8] Martinez MC, Iwamoto VE, Latorre Mdo R, Noronha AM, Oliveira AP, Cardoso CE (2016). Transcultural adaptation of the Johns Hopkins Fall Risk Assessment Tool. Rev Lat Am Enfermagem.

[9] Sousa LM, Marques-Vieira CM, Caldevilla MN, Henriques CM, Severino SS, Caldeira SM (2016). Risco de quedas em idosos residentes na comunidade: revisão sistemática da literatura. Rev Gaucha Enferm.

[10] Alexandre NM, Coluci MZ (2011). Validade de conteúdo nos processos de construção e adaptação de instrumentos de medidas. Cien Saude Colet.

[11] Fitzner K (2007). Reliability and validity: a quick review. Diabetes Educ.

[12] Organização Mundial da Saúde (OMS) (2004). Classificação Internacional de Funcionalidade Incapacidade de Saúde.

[13] Hernandez-Nieto R (2002). Contributions to statistical analysis.

[14] Balbinotti MA, Benetti C, Terra PR (2006). Translation and validation of the Graham-Harvey survey for the Brazilian context. Int J Manag Finance.

[15] Saldanha RP, Balbinotti MA, Balbinotti CA (2015). Tradução e validade de conteúdo do Youth Sport Value Questionnaire 2. Rev Bras Cien Esporte.

[16] Cassepp-Borges V, Teodoro ML (2007). Propriedades psicométricas da versão brasileira da Escala Triangular do Amor de Sternberg. Psicol Reflex Crit.

[17] Cassepp-Borges V, Balbinotti MA, Teodoro ML, Pasquali L (2010). Tradução e validação de conteúdo: uma proposta para a adaptação de instrumentos. Instrumentação psicológica: fundamentos e práticas.

[18] Balbinotti MA, Benetti C, Terra PR (2007). Translation and validation of the Graham-Harvey survey for the Brazilian context. Inter J Managerial Finance.

[19] Nakano TC, Siqueira LG (2012). Validade de conteúdo da Gifted Rating Scale (versão escolar) para a população Brasileira. Aval Psicol.

[20] Roediger MD, Marucci MF, Latorre MR, Hearst N, Oliveira C, Duarte YA (2017). Adaptação transcultural para o idioma português do método de triagem nutricional Determine your nutritional health^®^ para idosos domiciliados. Cien Saude Colet.

[21] Abreu DR, Azevedo RC, Silva AM, Reiners AA, Abreu HC (2016). Factors associated with recurrent falls in a cohort of older adults. Cien Saude Colet.

[22] Delbaere K, Gschwind YJ, Sherrington C, Barraclough E, Garrués-Irisarri MA, Lord SR (2016). Validity and reliability of a simple ‘low-tech’ test for measuring choice stepping reaction time in older people. Clin Rehabil.

[23] Chang ES, Beck T, Simon MA, Dong X (2014). A psychometric assessment of the psychological and social well-being indicators in the PINE study. J Aging Health.

[24] Ulian MD, Sato PM, Benatti FB, Campos-Ferraz PL, Roble OJ, Unsain RF (2017). Adaptação transcultural para o português dos Questionários de Desejos Intensos por Comida – Estado ou Traço (QDIC-E e QDIC-T) dos State and Trait Food-Cravings Questionnaires (FCQ-S and FCQ-T). Cien Saude Colet.

